# Do Optimists Like Vaccines? The Effect of Perceived Vaccine Novelty and Beliefs in the World’s Positivity and Orderliness on the Attitudes toward COVID-19 Vaccinations—The Case of European Young Adults

**DOI:** 10.3390/vaccines10030379

**Published:** 2022-03-01

**Authors:** Wojciech Trzebiński, Jerzy Trzebiński

**Affiliations:** 1 Department of Market, Marketing and Quality, Collegium of Management and Finance, SGH Warsaw School of Economics, 02-513 Warsaw, Poland; 2 Department of Psychology, SWPS University of Social Sciences and Humanities, 03-815 Warsaw, Poland; jerzy.trzebinski@swps.edu.pl

**Keywords:** COVID-19, vaccine attitudes, vaccination intent, vaccination advocacy, willingness to pay for vaccination, perceived vaccine effectiveness, perceived vaccine novelty, world’s orderliness/positivity beliefs, schema incongruity, consumer behavior

## Abstract

The public debate over COVID-19 vaccinations tends to focus on vaccine-related arguments, such as their effectiveness and safety. However, the characteristics of a person’s worldview, such as beliefs about the world’s positivity and orderliness, may also shape attitudes toward COVID-19 vaccinations. These relationships were investigated using schema incongruity theory. The degree of the vaccine’s incongruence with the world’s order schema existing in people’s minds was represented by perceived vaccine novelty. Accordingly, the results of an online survey among European young adults (*N* = 435) indicate that perceived vaccine novelty negatively affects behavioral outcomes (vaccination intent, willingness to pay for vaccinations, and vaccination advocacy). Moreover, there occurred a negative interaction effect of positivity and orderliness beliefs on behavioral outcomes. Specifically, an effect of positivity was more positive when people perceived the world as less ordered. Furthermore, this interaction effect was more negative when perceived vaccine novelty was higher. A mediating role of perceived vaccine effectiveness was demonstrated for the above relationships. The results extend the existing literature on people’s worldviews into the domain of vaccine attitudes, and provide new insights on the role of perceived vaccine novelty. For vaccination policymakers and marketers, the paper suggests how to promote vaccinations with consideration of orderliness/positivity beliefs and vaccine novelty perception.

## 1. Introduction

As vaccines and the process of vaccination may be treated as products, especially when paid for, public support for them can be viewed from a consumer behavior perspective. Favorable consumer attitudes toward vaccines take various forms: (a) the perception that vaccines are effective and safe [1,2], (b) intention to vaccinate [3], (c) willingness to pay for vaccinations [4], and (d) vaccination advocacy [5].

Despite the important role vaccinations may play in combating epidemics, vaccines remain controversial, pressing health policymakers and marketers to improve the effectiveness of vaccine advertising. For example, less than 67% of EU/EEA citizens were fully vaccinated against COVID-19 (with two doses) at the beginning of December 2021 [6], nearly one year after those vaccinations were made available. Among European populations, young adults may need a special advertising approach, as they perceive lower risks associated with COVID-19 [7,8].

Besides direct arguments about vaccines, such as vaccine effectiveness or safety, that prevail in social media discussions [9] and people’s declarations [10], attitudes toward vaccination may be shaped by people’s worldviews [11,12,13,14,15,16]. Therefore, considering such worldviews in vaccination advertising may enable marketers and policymakers to promote vaccines, at least partially bypassing the dispute over vaccines themselves.

As a factor in vaccination attitudes, this paper considers an important kind of optimism, rooted in people’s worldviews. Namely, crucial components of an optimistic worldview are belief in the world’s positivity and the world’s orderliness [17]. People differ in how strong these two beliefs are [18,19,20]. Our previous study [16] suggests that the belief in positivity may increase vaccination intent, but only if the belief in world orderliness is not firm. We speculated that this negative orderliness × positivity interaction effect results from viewing vaccines as standing beyond (outside) the world’s order. If the belief in that order is not firm, belief in the world’s positivity leads to a more positive attitude toward vaccinations. This mechanism was not further examined, but one may suppose that the orderliness × positivity interaction effect depends on perceived vaccine novelty, which is related to viewing vaccines as “intrusive“ and, thus, standing outside the world’s order. Although previous research provides indirect suggestions about the negative influence of perceived vaccine novelty on vaccination intent [21,22,23,24], the underlying psychological mechanism remains unexplored. Additionally, it is unknown how beliefs about orderliness and positivity and vaccine novelty shape related vaccine attitudes, such as perceived vaccine effectiveness, willingness to pay for vaccinations, and vaccination advocacy. Therefore, the current research poses the following question: How are various forms of vaccine attitudes (such as vaccination intent, willingness to pay for vaccination, vaccination advocacy, and perceived vaccine effectiveness) shaped by beliefs about the world’s orderliness/positivity and vaccine novelty?

## 2. Theoretical Background and Hypothesis Development

### 2.1. The Role of Beliefs about the World’s Orderliness and Positivity

People may have certain beliefs about the world’s orderliness, i.e., the degree to which the world works under regular patterns and rules, and the extent to which events are explainable and meaningful from a deeper perspective [19,20]. Likewise, people may form beliefs about the world’s positivity, i.e., the degree to which the world and people are good, trustworthy, and helpful. Those beliefs are positively correlated, and may be formed by early social experiences, such as the degree of predictability and caring in early relationships with parents [17,25,26,27]. Furthermore, the strength of these beliefs may change later as a result of exclusion from or inclusion into social groups [28], breakdown of long, intimate, and significant relationships [20,29], social anomie [30], and instability of social rules, high mortality, and poverty in macro-social environments [31,32]. These beliefs differ from ego-centered hope/optimism, which concerns mainly one’s future [33] or personal ability to achieve goals in a known circumstance [33,34,35]. However, the more unclear the future is, the more essential it is how the world is understood. Strong beliefs about the general order and positivity of the world lead to better outcomes after new life developments, such as trauma and significant and irreversible losses, or novel, unexpected opportunities and challenges [18,19]. The strengths of these two beliefs help overcome stress and trauma, and support post-traumatic growth. Perceiving the world as ordered and positive may also support life satisfaction, positive mood, and self-efficacy [36]. Constructs related to optimism and belief in the world’s orderliness/positivity, such as hope [37,38] and belief in a just world [39] are also present in consumer behavior studies.

In previous research, we explored the relationships between COVID-19 vaccination intent, perceived vaccine safety, and general beliefs about the world’s orderliness and positivity [16]. The authors studied Polish adult populations during the early stages of the pandemic. They consistently found a negative interaction effect between those two beliefs in two time periods (late 2020 and early 2021). Specifically, the effect of positivity on vaccination intent was more positive when people perceived the world as less ordered. We speculated that this negative interaction effect between the world’s orderliness and positivity beliefs is related to perceiving vaccinations as standing beyond (outside) the world’s order that is represented in people’s minds [16]. Namely, people may perceive vaccines as “intrusive” and breaking the “natural” chain of infections and curing. This is because vaccines are used as a preventive measure, rather than a response to the already contracted illness. In contrast, people may view illnesses and epidemics as natural and part of how the world is ordered. This perception may be particularly prominent in the case of COVID-19 vaccines, as they may be perceived as relatively rapidly invented, which, in turn, may contradict the typical view of incremental progress in medicine. Hence, when consumers perceive the world as highly ordered and positive, vaccines may appear unnecessary and even possibly harmful, as they do not belong to the positive order of the world. In this case, consumers may instead hope that the illness will not be harmful, or that some other means, more suitable to the world’s order, like a healthy lifestyle, would protect them from the worst consequences. Thus, when consumers believe more in a highly ordered world, belief in the world’s positivity may encourage less (or discourage more) reliance on vaccines. On the other hand, when consumers believe in a less-ordered world, a positive view of the world may encourage them to recognize important opportunities (such as an effective response to a contagious illness) outside of the world’s order. Then, vaccines, as not belonging to that order, may be appreciated as an effective tool.

To the best of our knowledge, the existing literature lacks other studies on the role of orderliness/positivity beliefs in vaccine attitudes. Hence, the mechanism proposed in our previous paper [16] needs to be further examined, specifically the negative interaction effect of these two beliefs on various forms of vaccine attitudes, such as perceived vaccine effectiveness, willingness to pay for vaccinations, and vaccination advocacy. This gap represents the practical dilemma of health policymakers and marketers designing vaccination advertisement campaigns: namely, whether and how one should promote vaccinations (i.e., make various forms of vaccine attitudes more favorable) with reference to the world’s orderliness and positivity. Furthermore, how could those favorable attitudes be formed across audiences with different orderliness/positivity beliefs? The current research aims to address this gap. Therefore, we pose the following question: How do beliefs about the world’s orderliness and positivity shape the various forms of vaccine attitudes?

We believe that the interpretation of the orderliness × positivity interaction effect formulated in our previous paper [16] aligns with the schema incongruity framework [40,41,42,43]. This theory posits that when a product is perceived or conceptualized as incongruent with the existing favorable and salient schema, and that incongruity is unresolved, consumers may form less-favorable attitudes toward that product. In our case, vaccines represent products, and the idea of the world’s order may be considered as the salient schema that exists in consumers’ minds. The assumption that vaccines are generally viewed as standing beyond (outside) the world’s order may reflect the unresolved incongruity between vaccines and the schema of the world’s order. Crucially, the favorability of the world’s order schema may depend on the interaction between the world’s orderliness and positivity beliefs. Namely, when the world is believed to be highly ordered and positive, the world’s order, as an essential component of the world, also has to be favorable. On the other hand, when belief in the world’s order is low, the order is an unimportant part of the world, and belief in the world’s positivity is less likely to increase a perceived favorability of the world’s order. In this case, belief in positivity may even encourage appreciating objects outside of the world’s order. Therefore, a positive interaction effect of orderliness and positivity may occur on the favorability of the world’s order schema. When assuming the general perception of vaccines as incongruent with the world’s order schema, it is reasonable to suppose that the higher favorability of this schema decreases positive vaccine attitudes. The latter, in turn, may explain the negative interaction effect of orderliness and positivity on vaccine attitudes.

**Figure 1:**
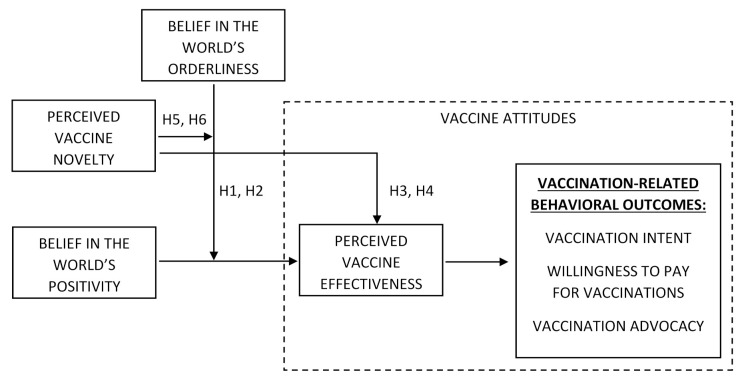
The conceptual model.

Perceived vaccine effectiveness is a form of vaccine attitude that may be directly affected by the vaccine/world schema incongruity. According to [16], given the belief in a highly ordered world, belief in the world’s positivity makes consumers hope that the world’s positive order (to which vaccines do not belong) can provide rescue from an epidemic. When people recognize a possible cause (in this case, the world’s positive order) of an event (in this case, relief from the epidemic), they tend to diminish the role of other causes (e.g., [44]), such as vaccine effectiveness. Therefore, a less-firm belief in the ordered world may make the belief in a positive world increase consumer hope in vaccine effectiveness more. Thus, we propose a negative interaction effect of belief in the world’s orderliness and positivity on perceived vaccine effectiveness. Therefore, we aim to test the following hypothesis (Figure 1):

**Hypothesis** **1** **(H1).** *There is a negative interaction effect of beliefs about the world’s positivity and orderliness on perceived vaccine effectiveness. Specifically, the effect of a belief in the world’s positivity on perceived vaccine effectiveness is more positive when consumer belief in the world’s orderliness is lower.*

Perceived vaccine effectiveness is a well-evidenced factor of vaccination intent (e.g., [45,46,47]), in line with the general notion that attitudes influence behavior [48]. Consequently, one may expect the negative orderliness/positivity interaction effect for other vaccination-related behaviors, such as paying for vaccination and vaccination advocacy. Formally (Figure 1):

**Hypothesis** **2** **(H2).** *The mediation between belief in the world’s positivity and vaccination-related behavioral outcomes, (that is, (a) vaccination intent; (b) willingness to pay for vaccination; and (c) vaccination advocacy) through perceived vaccine effectiveness is moderated at the first stage by belief in the world’s orderliness. Specifically, the indirect effects of positivity are more positive for lower levels of orderliness.*

The next step of our theorization investigates how the orderliness × positivity interaction effects discussed above may be strengthened by the degree to which people perceive vaccines as novel.

### 2.2. The Role of Perceived Vaccine Novelty

To further examine the mechanism of the negative orderliness × positivity interaction effect, we aim to conceptualize consumer perception of vaccines as standing beyond (outside) the world’s order. As discussed above, this perception may represent a case of incongruity between products (vaccines) and a mental schema of broader life domains such as human health, medicine, and illnesses. To this end, we refer to the concept of product novelty, which is widely used in consumer behavior literature. Novel products are defined as those whose attributes were changed based on modifications in technology [49]. Novelty may be related to the entire product category (e.g., [50]). In the case of vaccines, some consumers may perceive them as more novel, which may depend on various factors, such as health knowledge, orientation toward traditions and science, or education.

Consumers may prefer products perceived as novel in certain conditions, such as promotional (vs. preventive) regulatory focus, or hedonic product categories [51]. Novel product stimuli may also increase the positive effect of attitude towards advertisement on brand attitude [51]. On the other hand, perceived product novelty may lead to negative responses, such as disorientation and discord [49], or perceiving products as less usable [52]. Product novelty is considered in opposition to product familiarity, which is related to consumer experience with and ownership of a product [53]. Studies show a positive influence of perceived familiarity on adoption intentions for novel products. For example, adding familiar flavors to newly-developed meals may increase consumer evaluation [54].

Few studies have investigated the role of perceived vaccine novelty. For example, ref. [23] demonstrated a negative relationship between “COVID-19 vaccination adverse effects” and vaccination intent. The adverse effects were operationalized as negative consumer beliefs about the safety of vaccines, including an item directly related to novelty, i.e., “A coronavirus vaccination will be too new for me to be confident about getting vaccinated”. On the other hand, being vaccinated in the past was positively related to vaccination intent [21]. Likewise, people with higher vaccine familiarity were more likely to get vaccinated against COVID-19 [24]. It is noteworthy that even having an acquaintance who had been vaccinated was positively related to the acceptance of Hepatitis B vaccination [22].

Despite the above research efforts, which consistently, albeit indirectly, show that perceived novelty of (vs. familiarity with) vaccines may decrease vaccination intention, the interplay between perceived vaccine novelty and the effect of consumer orderliness/positivity beliefs on vaccine attitudes remains unknown. This gap is meaningful, because apart from the direct negative effect, vaccine novelty perception may play a more nuanced role in shaping vaccine attitudes. It corresponds to the practical issue of advertising vaccinations. Specifically, should one combine a reference to vaccine novelty and the world’s orderliness/positivity when promoting vaccinations? How should vaccination communication be aligned to audiences with different perceived vaccine novelty levels and orderliness/positivity beliefs? Therefore, we pose the question: How does perceived vaccine novelty, together with orderliness/positivity beliefs, shape vaccine attitudes?

We propose that higher perceived vaccine novelty (vs. familiarity) may correspond to perceiving vaccines as beyond (outside) the world’s order, and thus, represents an incongruity between the vaccine and the world’s order schema. Generally, perceived product novelty may produce incongruity versus existing consumer cognitive schemas, leading to negative emotional responses [49]. In the case of vaccines as a product category, perceiving them as novel may lead to viewing them as incongruent with general knowledge about how the world works. In other words, the more vaccines appear to be novel, the more they appear to stand beyond the world’s order. Assuming the schema of the world’s order as generally favorable (i.e., the world order is predominantly viewed as a good thing), this incongruity should harm vaccine attitudes. This way, those attitudes may be negatively influenced by perceived vaccine novelty. This idea appears to be in line with the empirical results of the aforementioned studies [21,22,23,24]. A vaccine’s novelty may not be as attractive as the novelty of other marketing products, such as novel car models or cosmetics, because vaccines may be perceived as deeply intervening in the human body and nature. Thus, the mental context of vaccines’ novelty may be more negative, as connected with “intrusiveness ”, rather than the positive aspects of civilization’s development. The novelty of COVID-19 vaccines, in particular, relates to the life-threatening pandemic: a new, unexpected event with a dynamic trajectory. Hence, perceiving vaccines as novel may make consumers more suspicious, resistant, and less confident about how vaccines work, e.g., how effective they are. In turn, decreased perception of vaccine effectiveness may negatively influence vaccination-related behavioral outcomes, such as vaccination intent, willingness to pay for vaccinations, and vaccination advocacy. Formally (Figure 1):

**Hypothesis** **3** **(H3).** *Vaccines are perceived as less effective when consumers perceive vaccines as more novel.*

**Hypothesis** **4** **(H4).** *Perceived vaccine effectiveness mediates the negative effect of perceived vaccine novelty on vaccination-related behavioral outcomes, that is, (a) vaccination intent, (b) willingness to pay for vaccinations, and (c) vaccination advocacy.*

As discussed above, the orderliness × positivity belief interaction may positively affect the favorability of the world’s order schema and, in turn, harm vaccine attitudes because of the incongruity between vaccines and the world’s order schema. In other words, a negative orderliness × positivity interaction effect on vaccine attitudes may result from perceiving vaccines as standing beyond the world’s order. Therefore, it is reasonable to suppose that the orderliness × positivity interaction effect on perceived vaccine novelty is enhanced (i.e., made more negative) by the degree of this incongruity, which is supposed to be perceived as vaccine novelty. Consequently, we aim to test the following hypothesis (Figure 1):

**Hypothesis** **5** **(H5).** *The interaction effect of orderliness/positivity beliefs on perceived vaccine effectiveness (H1) is more negative for consumers perceiving vaccines as more novel.*

Considering perceived vaccine effectiveness as a factor of vaccination-related behavioral outcomes, we further propose that perceived vaccine novelty moderates the first-stage moderation effect of orderliness in the mediation between positivity and the behavioral outcomes through perceived vaccine effectiveness. Formally (Figure 1):

**Hypothesis** **6** **(H6).** *Perceived vaccine novelty moderates the first-stage moderation effect of the world’s orderliness belief in the mediation between belief in the world’s positivity and vaccination-related behavioral outcomes (H2); that is, (a) vaccination intent, (b) willingness to pay for vaccination, and (c) vaccination advocacy, through perceived vaccination effectiveness. Specifically, the moderation effects of orderliness are more negative for higher levels of novelty.*

The hypotheses were tested in a web survey of European young adults during the COVID-19 pandemic, and vaccination attitudes were measured with reference to COVID-19 as a preventable illness.

## 3. Method

### 3.1. Procedure

Four hundred twenty-five European young adults (M_age_ = 22.3, SD = 2.8, 52.9% females, 84.0% studying, 34.1% working) participated in an online survey. The participants were recruited via electronic messaging by a team of thirty marketing research students, similar to [55], based on the following recruitment criteria: European country of residence, age 18–35, currently working or studying, at least high school level of education, and satisfactory English proficiency (see the sample characteristics in Appendix A). The study used homogenous, convenient sampling that is considered suitable for investigating general relationships between constructs (cf. [56]).

Data were collected during two weeks in October 2021, while about 1.98 mln new COVID-19 cases were detected in Europe, and 64.7% of the adult population in EU/EEA countries were fully vaccinated with two doses [6,57].

The questionnaire started with an instruction mentioning that whether the participants had (or had not) been vaccinated against COVID-19, they might still need to decide to vaccinate or not. The instruction continued with the following: “There are discussions about the next doses/shots. So suppose you are advised to get the COVID-19 vaccine”. This way, we aimed to gather consumer attitudes toward COVID-19 vaccinations (such as vaccination intent) even if a participant was already vaccinated. Next, we measured vaccine attitude constructs (vaccination intent, willingness to pay for vaccination, vaccination advocacy, and perceived vaccine effectiveness). The participants then rated vaccine novelty. Next, we measured beliefs about the world’s orderliness and positivity. We started the measurement with dependent variables to avoid self-generated validity issues [58,59]. In total, the questionnaire comprised twenty-three items measuring the studied constructs, followed by demographics.

### 3.2. Measurements

Except for the willingness to pay, all measurement scales (see details in Table 1) used seven-point response scales anchored with “definitely no” (1)/”definitely yes” (7). All vaccine attitude constructs (vaccination intent, willingness to pay for vaccinations, vaccination advocacy, and perceived vaccine effectiveness) were measured with respect to COVID-19 as a preventable illness.

Vaccination intent was measured with two items adapted from [3], α = 0.915. Vaccination advocacy was measured with three items adapted from [5], α = 0.933. The measurement of perceived vaccination effectiveness comprised two items (α = 0.870). The first one, adapted from [1], was related to protecting people from becoming seriously sick. The second item referred to keeping the pandemic under control, similar to [2]. We assessed unidimensionality across the three vaccine attitude constructs measured with measurement scales (i.e., vaccination intent and advocacy and perceived vaccine effectiveness). To this end, we ran exploratory factor analysis (EFA) with the fixed number of three factors (factors extracted using principal component analysis (PCA), Kaiser-Meyer-Olkin (KMO) test for sampling adequacy = 0.912, Bartlett’s *p* < 0.001). The factor with the highest eigenvalue represented 76.4% of the total variance, supporting unidimensionality. All items loaded heavily on the three rotated factors (loadings above 0.5; Varimax rotation) in line with the measurement scales. Three items showed cross-loadings of 0.4, but the differences to the loadings on the primary factors were above 0.2 [60]. The above results support the discriminant validity among the three vaccine attitude subdimensions.

Willingness to pay for vaccinations was measured in two steps, adapted from [4]. In the first step, the participants were asked whether they would pay 25 EUR for a vaccination (“yes/no”). The bid price in the second step depended on the response in the first step. If the answer was positive, the participants were asked whether they would pay 50 EUR (“yes/no”). If the answer in the first step was negative, the participants were asked whether they were willing to pay 12.5 EUR (“yes/no”). Willingness to pay was computed as the highest accepted price. In the case of negative answers in both steps, the willingness to pay was set to 0.

The measurement of perceived vaccine novelty (α = 0.730) started with the instruction “When you think about COVID-19 vaccination, your impression is that things of that kind …”, followed by two reversed items (“… have been done for a long time”, “… are well known”). They refer to being old-fashioned and conventional, as in the novelty measurement used by [61].

Belief in the world’s positivity was measured with five items [18,62]. Belief in the world’s orderliness was measured with seven items [18,62], one of which (“There is some unshakable world order that even the greatest catastrophes and changes cannot threaten”) was dropped, as it lowered the scale reliability.

To assess the discriminant validity for all measurements (except for the willingness to pay), we subjected the items from all respective measurement scales to EFA (factors extracted using PCA, KMO test for sampling adequacy = 0.894, Bartlett’s *p* < 0.001), which produced four components with eigenvalues above 1. The factor with the highest eigenvalue represented 27.5% of the total variance, indicating no issue with common method bias [63]. A factor loading cut-off of 0.5 was applied (Varimax rotation). For the four factors, the heavily-loaded items belonged to (1) vaccine attitude scales (i.e., vaccination intent and advocacy, and perceived vaccine effectiveness), (2) the perceived vaccine novelty scale, (3) the world’s orderliness belief scale, and (4) the world’s positivity belief scale. Five items showed cross-loadings of 0.4, but the differences to the loadings on the primary factors were above 0.2. However, we decided to drop four of those items. The first three dropped items belonged to the orderliness measurement, but they contained some positivity aspects (i.e., “The world is fair, and sooner or later, everyone will get what someone deserves”, “The world is meaningful, and all things have some sense even if we feel lost sometimes”, “Righteous and good people will be rewarded in some way”). The reliability of the reduced orderliness scale was α = 0.698. The fourth dropped item belonged to the positivity measurement, but it might contain an orderliness aspect as it referred to the ultimate result of things that would happen (“Evil, which is so much around but ultimately will not triumph over good”). The reliability of the reduced positivity scale was α = 0.756. In sum, the EFA results support the discriminant validity between the final measurements.

Apart from the willingness to pay, the measurement scale items were averaged into single indices for all constructs. We tested the hypotheses based on ordinary least squares (OLS) regressions in PROCESS macro [64], as it is suitable for analyzing interactions between continuous variables (cf. [65]), which are essential in our model.

## 4. Results

### 4.1. Testing H1 and H2 (the Role of Beliefs about the World’s Orderliness and Positivity)

**Figure 2:**
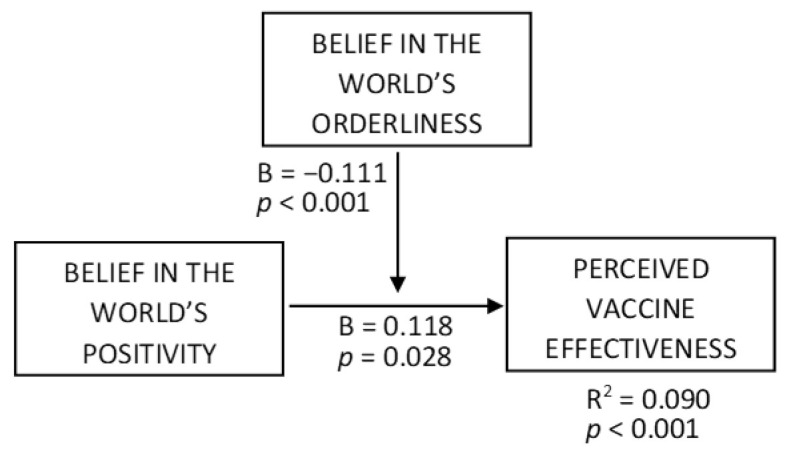
Moderation effects of belief in the world’s orderliness on the relationship between belief in the world’s positivity and perceived vaccine effectiveness.

**Figure 3:**
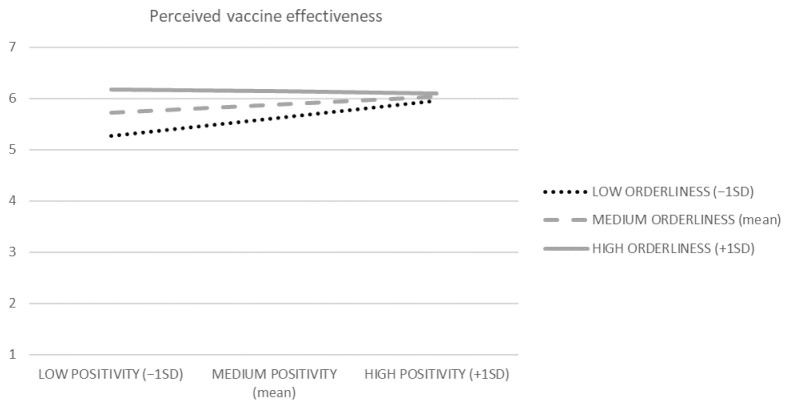
Visualization of the conditional effects of belief in the world’s positivity on perceived vaccine effectiveness (belief in the world’s orderliness is the moderator).

H1 predicted a negative interaction effect of beliefs about the world’s orderliness and positivity on perceived vaccination effectiveness. To test this, we ran a moderation analysis (PROCESS macro, Model 1), where positivity served as an independent variable, orderliness was a moderator, and perceived vaccination effectiveness was a dependent variable (Figure 2, R^2^ = 0.090, F = 13.873, *p* < 0.001, variance inflation factors (VIFs) < 1.2). The orderliness × positivity interaction effect was negative (B = −0.111, t = 3.356, *p* < 0.001). The conditional effect of positivity at the orderliness level of −1SD was positive (Figure 3, B = 0.263, t = 3.733, *p* < 0.001), while being non-significant at the +1SD orderliness level (*p* > 0.6). Thus, H1 is supported.

H2 predicted moderated mediation, with perceived vaccine effectiveness as a mediator of the positivity-behavior relationships, and orderliness as a first-stage moderator. Specifically, the moderated mediation indices were expected to be negative. To test it, we ran a series of moderated mediation analyses (PROCESS macro, Model 7, 5000 bootstrap samples). Belief in the world’s positivity served as an independent variable, belief in the world’s orderliness was the first-stage moderator, and perceived vaccination effectiveness was a mediator (VIFs < 1.3), and vaccination-related behavioral outcomes were dependent variables. Below, we present those analyses for each behavioral construct.

**Figure 4:**
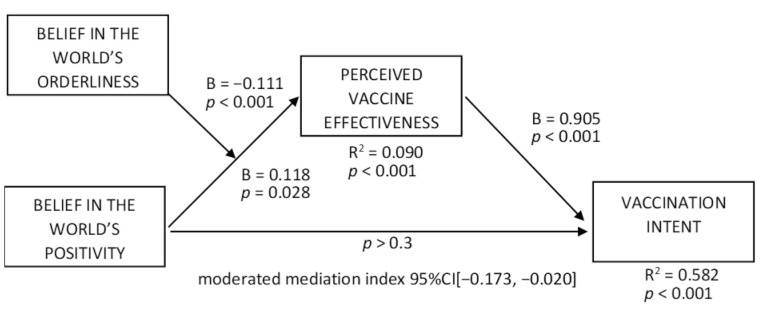
Moderated mediation effects on vaccination intent.

To test H2a, we ran the moderated mediation model with vaccination intent as a dependent variable (Figure 4); the effect of the perceived vaccine effectiveness was positive (B = 0.905, t = 23.975, *p* < 0.001; R^2^ = 0.582, *p* < 0.001), and the moderated mediation index was negative (95% CI [−0.173, −0.020]). The conditional indirect effect of the positivity belief on vaccination intent at the orderliness level of −1SD was positive (95% CI [0.060, 0.393]), while it was non-significant at the mean and +1SD orderliness levels. Those results support H2a.

To test H2b, we ran the moderated mediation model with the willingness to pay for vaccinations as a dependent variable; the effect of the perceived vaccine effectiveness was positive (B = 7.870, t = 12.505, *p* < 0.001; R^2^ = 0.271, *p* < 0.001), and the moderated mediation index was negative (95% CI [−1.490, −0.199]). The conditional indirect effect of positivity belief on willingness to pay at the orderliness level of −1SD was positive (95% CI [0.543, 3.518]), while it was non-significant at the mean and +1SD orderliness levels. Those results support H2b.

To test H2c, we ran the moderated mediation model with vaccination advocacy as a dependent variable; the effect of perceived vaccine effectiveness was positive (B = 1.035, t = 26.868, *p* < 0.001; R^2^ = 0.639, *p* < 0.001), and the moderated mediation index was negative (95% CI [−0.189, −0.024]). The conditional indirect effect of the positivity belief on vaccination advocacy at the orderliness level of −1SD was positive (95% CI [0.070, 0.444]), while it was non-significant at the mean and +1SD orderliness levels. Those results support H2c.

### 4.2. Testing H3 and H4 (the Effects of Perceived Vaccine Novelty on Vaccine Attitudes)

To test H3 (predicting the positive effect of perceived vaccine novelty on perceived vaccine effectiveness) and H4 (predicting the mediating role of perceived vaccine effectiveness on the relationship between perceived vaccine novelty and vaccination-related behavioral outcomes), we ran a series of mediation analyses (PROCESS macro, Model 4, 5000 bootstrap samples). Perceived novelty was an independent variable, perceived effectiveness was a mediator, and the behavioral outcomes were dependent variables.

**Figure 5:**
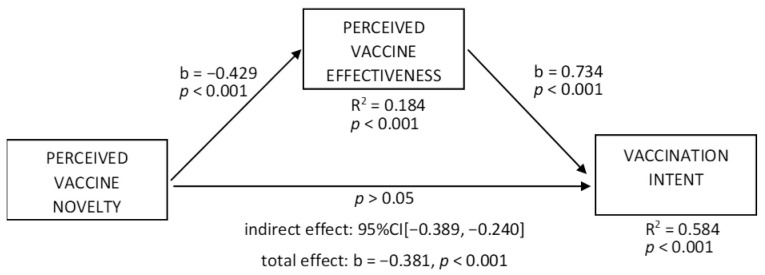
Mediation effect of perceived vaccine effectiveness—vaccination intent as a dependent variable.

To test H3 and H4a, we ran the mediation model with vaccination intent as a dependent variable (Figure 5); the effect of perceived vaccine novelty on perceived vaccine effectiveness was negative (b = −0.429, t = 9.74, *p* < 0.001; R^2^ = 0.584, *p* < 0.001), supporting H3. Novelty had a negative total effect on vaccination intent (b = −0.381, t = 8.469, *p* < 0.001); perceived vaccine effectiveness showed a positive effect on vaccination intent (b = 0.734, t = 21.126, *p* < 0.001), and the indirect effect of novelty was negative (95% CI [−0.389, −0.240]), supporting H4a. Moreover, the direct effect was non-significant, suggesting full mediation.

To test H4b, we ran the mediation model with willingness to pay for vaccinations as a dependent variable; novelty had a negative total effect on willingness to pay (b = −0.306, t = 6.618, *p* < 0.001; R^2^ = 0.278, *p* < 0.001), perceived vaccine effectiveness showed a positive effect on willingness to pay (b = 0.472, t = 10.294, *p* < 0.001), and the indirect effect of novelty was negative (95% CI [−0.263, −0.148]), supporting H4b.

To test H4c, we ran the mediation model with vaccination advocacy as a dependent variable; novelty had a negative total effect on vaccination advocacy (b = −0.449, t = 10.213, *p* < 0.001; R^2^ = 0.652, *p* < 0.001), perceived vaccine effectiveness showed a positive effect on vaccination advocacy (b = 0.746, t = 23.439, *p* < 0.001), and the indirect effect of novelty was negative (95% CI [−0.389, −0.252]), supporting H4c.

### 4.3. Testing H5 and H6 (the Moderating Role of Perceived Vaccine Novelty)

**Figure 6:**
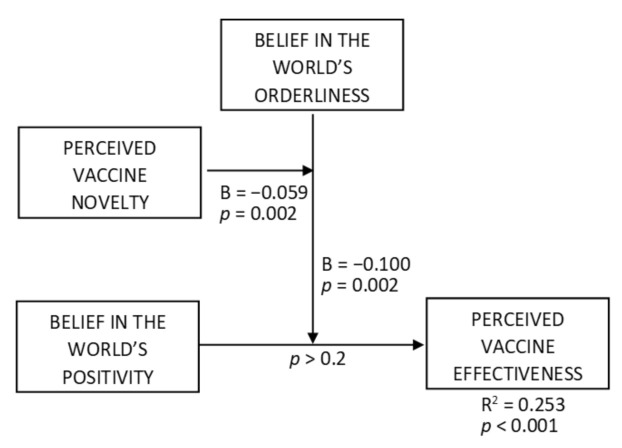
Moderated moderation effect of perceived vaccine novelty and belief in the world’s orderliness on the relationship between belief in the world’s positivity and perceived vaccine effectiveness.

**Figure 7:**
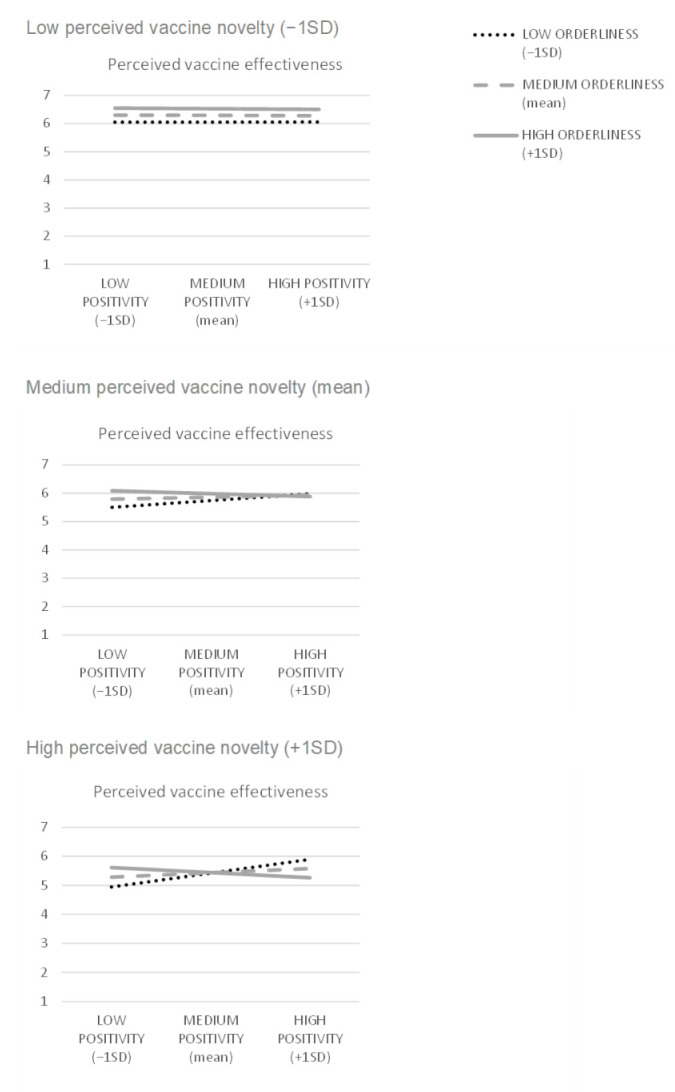
Visualization of conditional effects of belief in the world’s positivity on perceived vaccine effectiveness (belief in the world’s orderliness and perceived vaccine novelty are the moderators).

H5 predicted that the interaction effect of beliefs about the world’s orderliness and positivity on perceived vaccine effectiveness is more negative for consumers who perceive vaccines as more novel. To test this, we ran a moderated moderation analysis (PROCESS macro, Model 3), where the positivity belief served as an independent variable, the orderliness belief was the first moderator (moderating the positivity-effectiveness relationship), perceived vaccine novelty was a second moderator (moderating the moderation) (VIFs < 1.5), and perceived vaccine effectiveness was a dependent variable (Figure 6, R^2^ = 0.253, F = 20.122, *p* < 0.001). The effect of perceived vaccine novelty on perceived vaccine effectiveness was negative (B = −0.274, t = 6.578, *p* < 0.001), as was the novelty × orderliness × positivity interaction effect (B = −0.100, t = 3.148, *p* < 0.001). The orderliness × positivity interaction effect was also negative (B = −0.059, t = 3.764, *p* = 0.002). Both novelty × orderliness and novelty × positivity interaction effects were non-significant (*p*_novelty × positivity_ > 0.2, *p*_novelty × orderliness_ > 0.06). The conditional orderliness × positivity interaction effects at the mean and +1SD novelty levels were negative (B_mean novelty_ = −0.100, F = 9.912, *p* = 0.002; B_+1SD novelty_ = −0.192, F = 22.044, *p* < 0.001), while being non-significant at the −1SD novelty level (*p* > 0.8). Among the conditional effects of positivity at −1SD, mean, and +1SD levels of orderliness and novelty (Figure 7), the positive effects occurred for the −1SD orderliness level combined with the mean and the +1SD levels of novelty (B_mean novelty_ = 0.185, t = 2.774, *p* = 0.006; B_+1SD novelty_ = 0.364, t = 4.426, *p* < 0.001), while being non-significant for other levels of the moderators. These results support H5.

H6 predicted moderated moderated mediation, with perceived vaccine effectiveness as a mediator of positivity-behavior relationships, orderliness as a first-stage moderator of those mediations, and novelty as a moderation moderator. Specifically, the moderated mediation indices were expected to be more negative for higher levels of perceived vaccine novelty. To test it, we ran a series of moderated moderated mediation analyses (PROCESS macro, Model 11, 5000 bootstrap samples). Belief in the world’s positivity was an independent variable, belief in the world’s orderliness was the first moderator (moderating the mediation), perceived vaccine novelty was a second moderator (moderating the moderation), perceived vaccination effectiveness was a mediator (VIFs < 1.5), and the vaccination-related behavioral outcomes were dependent variables. Below, we present those analyses for each behavioral construct.

**Figure 8:**
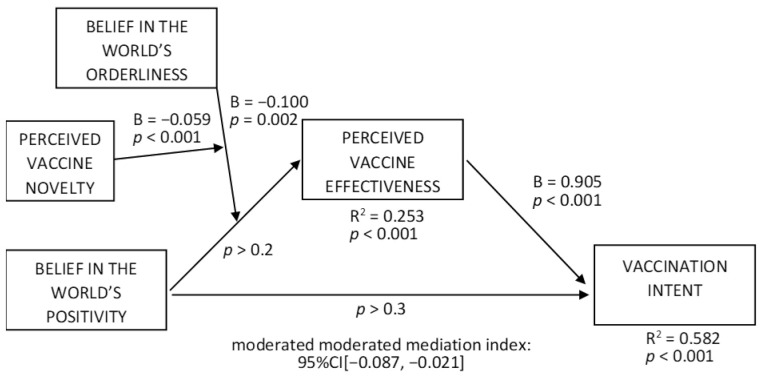
Moderated moderated mediation effects on vaccination intent.

To test H6a, we ran the moderated moderated mediation model with vaccination intent as a dependent variable (Figure 8); the effect of perceived vaccine effectiveness was positive (B = 0.905, t = 23.975, *p* < 0.001; R^2^ = 0.582, *p* < 0.001), and the moderated moderated mediation index was negative (95% CI [−0.087, −0.021]). The conditional indices of moderated mediation (orderliness as a moderator) were negative at the mean and +1SD levels of perceived vaccine novelty (95% CI [−0.162, −0.016], 95% CI [−0.267, −0.080], respectively) while being non-significant at the −1SD novelty level. Among the conditional indirect effects of positivity on vaccination intent at −1SD, mean, and +1SD levels of orderliness and novelty, the positive effects occurred for the −1SD orderliness level combined with the mean and +1SD levels of novelty (for mean novelty: 95% CI [0.005, 0.311]; for +1SD novelty: 95% CI [0.132, 0.505]) while being non-significant for other levels of the moderators. These results support H6a.

To test H6b, we ran the moderated moderated mediation model with the willingness to pay for vaccinations as a dependent variable; the effect of perceived vaccine effectiveness was positive (B = 7.870, t = 12.505, *p* < 0.001; R^2^ = 0.271, *p* < 0.001), and the moderated moderated mediation index was negative (95% CI [−0.747, −0.203]). The conditional indices of moderated mediation (orderliness as a moderator) were negative at the mean and +1SD levels of perceived vaccine novelty (95% CI [−1.377, −0.112], 95% CI [−2.280, −0.715], respectively), while being non-significant at the −1SD novelty level. Among the conditional indirect effects of positivity on willingness to pay at −1SD, mean, and +1SD levels of orderliness and novelty, positive effects occurred for the −1SD orderliness level combined with the mean and +1SD levels of novelty (for mean novelty: 95% CI [0.018, 2.724]; for +1SD novelty: 95% CI [1.080, 4.445]), while being non-significant for other levels of the moderators. Thus, H6b was also supported.

To test H6c, we ran the moderated moderated mediation model with vaccination advocacy as a dependent variable; the effect of perceived vaccine effectiveness was positive (B = 1.035, t = 26.858, *p* < 0.001; R^2^ = 0.639, *p* < 0.001), and the moderated moderated mediation index was negative (95% CI [−0.098, −0.025]). The conditional indices of moderated mediation (orderliness as a moderator) were negative at the mean and +1SD levels of perceived vaccine novelty (95% CI [−0.179, −0.017], 95% CI [−0.298, −0.093], respectively), while being non-significant at the −1SD novelty level. Among the conditional indirect effects of positivity on vaccination advocacy at −1SD, mean, and +1SD levels of orderliness and novelty, positive effects occurred for the −1SD orderliness level, combined with the +1SD level of novelty (95% CI [0.140, 0.568]), while being non-significant for other levels of the moderators. These results support H6c.

## 5. Discussion

In line with our expectations, beliefs about the world’s orderliness and its positivity showed a negative interaction effect on perceived vaccine effectiveness. The effectiveness, in turn, appeared to transfer this interaction effect to the behavioral outcomes of vaccination intent, willingness to pay for vaccinations, and vaccination advocacy. Specifically, the results suggest that belief in the world’s orderliness diminishes the positive effect of belief in the world’s positivity on vaccine attitudes. Moreover, we demonstrated the role of perceived vaccine novelty in the orderliness × positivity interaction effect. Namely, the results suggest that perceived vaccine novelty enhances the degree to which belief in orderliness diminished the positive positivity effect on vaccine attitudes. Finally, perceived vaccine novelty appears to influence perceived vaccination effectiveness negatively, and in turn, harm the behavioral outcomes.

Our results suggest that an optimistic worldview in the form of a strong belief in the world’s positivity leads consumers to accept vaccinations, especially when the world is viewed as not ordered (disordered) and vaccines are perceived as highly novel. We demonstrated the mechanism behind those interaction effects. Specifically, the current results provide support for our previous speculation [16] that people generally perceive vaccines as standing beyond (outside) the world’s order, and subsequently, a strong belief in the world’s order (or disorder) diminishes (or enhances) the degree to which belief in the world’s positivity makes consumers appreciate the vaccines. First, we demonstrated the orderliness × positivity interaction effect on the mediation relationship between positivity and vaccination-related behavioral outcomes through perceived vaccine effectiveness. Second, we showed the moderation effect of perceived novelty (which may represent perceiving vaccines as standing beyond the world’s order) on the orderliness × positivity interaction effect. We also integrated the above findings in moderated moderated mediation models. It is noteworthy that our study involves additional vaccine attitude constructs compared to our previous research [16]: perceived vaccine effectiveness, willingness to pay for vaccination, and vaccination advocacy. By proposing the mechanism by which the world’s orderliness/positivity beliefs shape vaccine attitudes, we add to the broad literature on the consequences of optimism and orderliness/positivity beliefs [20,25,26,27,36,37,38,39]. Furthermore, we extend this literature into the domain of vaccine attitudes by suggesting the moderator (perceived vaccine novelty) of orderliness/positivity effects.

Our research contributes to vaccine perception knowledge by demonstrating the negative effect of perceived vaccination novelty on vaccine attitudes. This finding is in line with previous studies [21,22,23,24]; however, the above studies relate to vaccine novelty perception rather indirectly, as being vaccinated in the past [21], knowing someone vaccinated [22], and declared knowledge about vaccines [24]. Sherman et al. [23] referred to perceiving vaccinations as “too new for me”, which represents the perceived novelty more directly, but it was involved only as one item in the measurement scale of perceived “vaccination adverse effects”. Moreover, all the above studies focus on vaccines from a personal perspective on vaccines (such as personal experience, friends, or knowledge), while we considered perceived novelty as a view on vaccines as being in long-term use and well-known by people in general. Additionally, while the previous studies focused on vaccination intent, we involved other forms of vaccine attitudes (perceived vaccine effectiveness, willingness to pay for vaccinations, and vaccination advocacy), and demonstrated the mediating role of effectiveness in the relationship between novelty and vaccination-related behavioral outcomes.

Finally, our research adds to the rich literature based on the schema incongruity framework [40,41,42,43]. Namely, we consider perceived vaccine novelty as representing the incongruity between vaccines and the schema of the world’s order. Moreover, we propose the interaction effect of the world’s orderliness and positivity beliefs on the favorability of the world’s order schema. Our findings show that this novel approach explains the relationships among vaccine attitudes, perceived vaccine novelty, and orderliness/positivity beliefs.

## 6. Conclusions

This research aimed to investigate the role of peoples’ worldviews on attitudes toward COVID-19 vaccines. The results suggest that the impact of beliefs about the world’s orderliness and positivity on those attitudes depends on perceiving vaccines as novel (“intrusive” versus the well-known world). This way, perceived vaccine novelty was considered as an indicator of a degree of incongruity between the vaccines and the schema of the world’s order. The implications of the current results are summarized in Table 2.

### 6.1. Possible Applications in Vaccine Communication

The proposed mechanism (i.e., the positive effect of belief in the world’s positivity on vaccine attitudes, with perceived vaccine novelty and belief in the world’s orderliness as moderators) implies several practical applications for vaccination policymakers and marketers. This is especially vital for COVID-19 vaccines, as the pandemic may evolve into the endemic stage, and favorable vaccine attitudes are essential to maintain (or enhance) the achieved levels of vaccination in societies. Our results suggest that besides arguments directly related to vaccines (such as their effectiveness or safety), vaccination-promoting advertisements may refer to consumer worldview in the form of beliefs about the world’s positivity and orderliness. Those beliefs may be treated as personal characteristics or ideas able to be activated. Thus, vaccination advertising can target consumers having specific positivity/orderliness beliefs (e.g., via social media) or activate those beliefs. For example, if a targeted consumer perceives vaccines as highly novel and the world as rather disordered, the positive effect of the positivity belief on vaccine attitudes is likely. Therefore, in this case, vaccination promoters may wish activate the idea of the world’s positivity while encouraging the consumer to vaccinate. One may consider a message such as: “The world is good! It is worth taking advantage of vaccines”.

Next, vaccination campaigns can emphasize that vaccines are not novel, as reducing perceived vaccine novelty may make vaccination attitudes more favorable. An exemplary message could be: “Vaccines have been with people for a long time”. An alternative approach may be better when targeted consumers firmly believe that vaccines are novel. The campaigns can then focus on building positive associations with vaccine novelty, which may help to resolve the incongruity between vaccines and the world’s order schema. An illustrative message following this strategy can be: “Novelty is good! It is worth looking for new opportunities, even if they go beyond what is well-known to us”.

Generally, as our research highlights the influence of consumer worldview on vaccine attitudes, we encourage vaccination policymakers and marketers to engage endorsers who a target group may perceive as having a similar worldview (e.g., former anti-vaxxers who changed their mind).

### 6.2. Limitations and Future Research Directions

The current study was based on homogenous and convenient sampling, which is appropriate to investigate the general relationships between constructs, but does not provide a representative sample for a given population. Our sample confirms the diversity of vaccine attitudes across European countries, supporting previous findings [66]. For example, our Portuguese participants showed more positive vaccine attitudes (vaccination intent, willingness to pay for vaccinations, vaccination advocacy, and perceived vaccine effectiveness) compared to our Polish participants (all *p*s < 0.01). However, country-to-country comparison is possible only for some countries with a sufficient number of participants in the sample. Therefore, future research based on representative samples needs to involve vaccine attitudes and worldview constructs, such as beliefs about the world’s orderliness and positivity. This way, the relationships evidenced in the current study could be validated across various demographic variables. Specifically, vaccine attitudes and beliefs about the world’s orderliness/positivity may be compared across countries.

The current research is correlational, so our results need to be further examined experimentally through the activation of the idea of the world’s positivity in vaccination advertisements. Importantly, different beliefs about the world’s orderliness and vaccine novelty should be considered. The proposed effects on vaccine attitudes may also be observed in field studies dealing with consumer responses to actual advertisement campaigns. Those effects should be tested in future stages of the COVID-19 pandemic and for other preventable illnesses.

Our results are inconclusive in terms of the influence of belief in the world’s orderliness on vaccine attitudes. In our dataset, the effects of a belief in orderliness on vaccine attitudes were positive (see Figure 3), while in our previous research [16], neutral or slightly negative effects occurred. Perhaps, the positive effect of belief in orderliness, visible in the current study, occurred because that belief might lead to viewing the world’s order as more extensive, thus embracing vaccines. Consequently, the orderliness might decrease perceived vaccine novelty and, in turn, increase vaccine attitudes. Accordingly, the effects of orderliness beliefs on vaccine attitudes were mediated by perceived vaccine novelty in our dataset. Interestingly, our Portuguese participants (who were more positively oriented towards vaccinations, as mentioned above) show a marginally higher level of belief in the world’s orderliness than our Polish participants (*p* = 0.07). It may preliminarily suggest that the orderliness belief, perhaps through the mechanism discussed above, may explain differences in vaccine attitudes across countries. On the other hand, the negative effect of belief in orderliness, reported in our previous paper [16], might occur because that belief might make the schema of the world’s order more salient, increasing the negative impact of the incongruity between vaccines and the world’s order on vaccine attitudes (cf. [43]). Future studies may attempt to examine those two antagonistic mechanisms and identify their moderators or boundary conditions, also using representative samples that would allow country-level comparisons.

The current research does not study incongruity resolution as a separate factor. The schema incongruity framework posits that incongruity’s negative influence on attitudes diminishes when the incongruity is resolved [40,43]. Thus, it is expected that vaccine attitudes can be improved when the discrepancy between vaccines and the world’s order is explained to consumers. Future research should explore this opportunity.

Finally, our research does not investigate the factors of perceived vaccine novelty. It may depend on worldviews (such as traditional values). Investigating this may extend the understanding of the worldview context for the role of vaccine novelty in shaping vaccine attitudes.

## Figures and Tables

**Table 1:** The final measurement scales.

Construct	Item	Reference	Reliability
Vaccination intent	I intend to get vaccinated for COVID-19.	Adapted from [3]	α = 0.915
	If the COVID-19 vaccine is free, I would like to get the vaccine.		
Vaccination advocacy	I am willing to encourage others to get vaccinated.	Adapted from [5]	α = 0.933
	I am willing to provide arguments in favor of the vaccination.		
	I am willing to defend the vaccines if someone criticizes them.		
Perceived vaccine effectiveness	COVID-19 vaccines are effective in protecting people from becoming seriously sick with COVID-19.	Adapted from [1]	α = 0.870
	If most people vaccinate against COVID-19, the pandemic will be kept under control or defeated.	Similar to [2]	
Perceived vaccine novelty	When you think about COVID-19 vaccination, your impression is that things of that kind have been done for a long time.	Inspired by [61]	α = 0.730
	When you think about COVID-19 vaccination, your impression is that things of that kind are well known.		
Belief in the world’s positivity	There will always be some people who will help us in a misfortune.	Adapted from [18,62]	α = 0.756
	People can be trusted.		
	The world is good even if we are not doing well.		
	People are good by nature.		
Belief in the world’s orderliness	All that happens in the world is or can be explainable.	Adapted from [18,62]	α = 0.698
	The world is governed by general regularities, although it is sometimes difficult to see and understand them.		
	Looking from a deeper perspective, even the most incomprehensible paths of human life are explainable and have some sense.		

**Table 2:** Overview of the results and implications.

Hypothesis Testing	Theoretical Implications/Contributions		Practical Implications (Examples)
H1. The positive effect of belief in the world’s positivity on perceived vaccination effectiveness is weaker when consumer belief in the world’s orderliness is higher. SUPPORTED	Provides evidence for the negative orderliness × positivity interaction effect on vaccine attitudes [16], involving the perceived vaccine effectiveness as a mediator and other behavioral outcomes (i.e., the willingness to pay and vaccination advocacy).	Extends the literature on the consequences of optimism [20,25,26,27,36,37,38,39] into the domain of vaccine attitudes.	If a targeted consumer perceives vaccines as highly novel and the world as rather disordered, the vaccination advertisements can activate the idea of the world’s positivity.
H2. Belief in the world’s orderliness moderates (at the first stage) the mediation between belief in the world’s positivity and the vaccination-related behavioral outcomes through perceived vaccination effectiveness. Specifically, the indirect effects of positivity are weaker (less positive) for higher levels of orderliness. SUPPORTED			
H5. The negative interaction effect of orderliness/positivity beliefs on perceived vaccine effectiveness (H1) is stronger for consumers perceiving vaccines as more novel. SUPPORTED	Provides further evidence for the negative orderliness × positivity interaction effect on vaccine attitudes [16], involving the perceived vaccine novelty as a moderator.		
H6. Perceived vaccine novelty moderates the first-stage moderation effect of belief in the world’s orderliness on the mediation between belief in the world’s positivity and the vaccination-related behavioral outcomes (H2) through perceived vaccination effectiveness. Specifically, the moderation effects of orderliness are stronger (more negative) for higher levels of novelty. SUPPORTED			
H3. Vaccines are perceived as less effective when consumers perceive vaccines as more novel. SUPPORTED	Extends previous studies on vaccination intent [21,22,23,24] by directly demonstrating the effects of perceived vaccine novelty on vaccination-related behavioral outcomes and demonstrating the mediating role of perceived vaccine effectiveness in those effects.		Vaccination advertisements can emphasize that vaccines are not novel or build positive associations with vaccine novelty.
H4. Perceived vaccine effectiveness mediates the negative effect of perceived vaccine novelty on vaccination-related behavioral outcomes. SUPPORTED			

## Data Availability

The data presented in this study are available on request from the corresponding author.

## Appendix A

**Table A1:** The Sample Characteristics.

	Frequency	Percentage
**Gender**		
Women	225	52.9
Men	193	45.4
Other/no response	7	1.6
**Age**		
under 20	25	5.9
20–24	340	80.0
25–29	43	10.1
30 or more	17	4.0
**Education**		
High school	234	55.1
University	191	44.9
**Occupation**		
Only studying	280	65.9
Only working	68	16.0
Working and studying	77	18.1
**Previous vaccination against COVID-19**		
Vaccinated	368	86.6
Unvaccinated	47	11.1
No response	10	2.4
**Country of residence**		
Belarus	7	1.6
Belgium	4	0.9
Czechia	2	0.5
Denmark	2	0.5
France	7	1.6
Georgia	16	3.8
Germany	84	19.8
Greece	12	2.8
Ireland	2	0.5
Italy	55	12.9
Lithuania	1	0.2
Poland	91	21.4
Portugal	56	13.2
Russia	1	0.2
Spain	68	16.0
Switzerland	4	0.9
Netherlands	5	1.2
United Kingdom	4	0.9
Ukraine	1	0.2
No response	3	0.7
